# PSO-XnB: a proposed model for predicting hospital stay of CAD patients

**DOI:** 10.3389/frai.2024.1381430

**Published:** 2024-05-03

**Authors:** Geetha Pratyusha Miriyala, Arun Kumar Sinha

**Affiliations:** School of Electronics Engineering, VIT-AP University, Amaravati, Andhra Pradesh, India

**Keywords:** coronary artery disease, length of stay prediction, uncertainty, deep autoencoder, boosting algorithms, particle swarm optimization

## Abstract

Coronary artery disease poses a significant challenge in decision-making when predicting the length of stay for a hospitalized patient. This study presents a predictive model—a Particle Swarm Optimized-Enhanced NeuroBoost—that combines the deep autoencoder with an eXtreme gradient boosting model optimized using particle swarm optimization. The model uses a fuzzy set of rules to categorize the length of stay into four distinct classes, followed by data preparation and preprocessing. In this study, the dimensionality of the data is reduced using deep neural autoencoders. The reconstructed data obtained from autoencoders is given as input to an eXtreme gradient boosting model. Finally, the model is tuned with particle swarm optimization to obtain optimal hyperparameters. With the proposed technique, the model achieved superior performance with an overall accuracy of 98.8% compared to traditional ensemble models and past research works. The model also scored highest in other metrics such as precision, recall, and particularly F1 scores for all categories of hospital stay. These scores validate the suitability of our proposed model in medical healthcare applications.

## 1 Introduction

Nearly 31% of all deaths around the globe are due to cardiovascular diseases, among which coronary artery disease (CAD) is the most common. By 2030, an estimated 22 million people will likely be affected by CAD (Workina et al., 2022). The risk factors causing CAD are inactivity, smoking, excessive alcohol consumption, poor diet, and a sedentary lifestyle (Balen et al., 2024). To determine the patient mortality risk in the health sector, clinicians often consider the patient's length of stay (LOS) as a critical indicator (Awad et al., 2017). The statistics show that approximately 33% of older adult patients admitted to intensive care units (ICUs) due to prolonged LOS do not survive (Li et al., 2024). Most statistical analysis and current research solely focus on predicting the overall target LOS or singular LOS class, i.e., short or long stay. This limits the ability to determine the important prediction insights of each hospital stay duration, showing a significant research gap. Therefore, to address this gap, our study focuses on four distinct LOS classes: short, medium, long, and extended stays (Abdurrab et al., 2024; Junior et al., 2024; Momo et al., 2024). A short stay typically signifies quick recoveries, though it may sometimes indicate early mortality. Whereas long stay often indicates the presence of severe health issues or long-term illnesses. On the other hand, extended stay refers to prolonged hospital stay due to severe illness (Heyland et al., 2015). Meanwhile, a medium stay indicates ongoing treatment or monitoring of moderate medical issues.

In the medical sector, the LOS plays a crucial role in managing various health conditions such as diabetes (Ata et al., 2023), cancer (Jung et al., 2023), tumors (Alzubi et al., 2019; Muhlestein et al., 2019), chronic kidney disease (Neyra et al., 2023), inflammatory conditions (Mangalesh et al., 2023), and infectious diseases (Saadatmand et al., 2023). The accurate prediction of LOS at the preliminary stage can aid clinicians and patients in decision-making about treatment and recovery planning, resource, and budget allocation and help patients reduce mortality rate (Asadi-Lari et al., 2004). Therefore, it highlights the need for a single, powerful machine-learning model to predict the LOS. Moreover, researchers use various artificial intelligence techniques to classify the LOS with automated decision-making systems (Masood et al., 2022). However, the challenge lies in dealing with the variability of data and the inherent uncertainties in predicting LOS. Therefore, with advanced machine learning (ML), deep learning (DL), comprehensive medical data, and fuzzy logic principles, our model aims to develop a robust classifier model capable of predicting LOS for CAD patients. This work proposes an enhanced modeling strategy with Particle Swarm Optimization (PSO) to improve accuracy. The main contributions of our research are:

- Developing a Particle Swarm Optimized-Enhanced NeuroBoost (PSO-XnB) model combining PSO with eXtreme Gradient Boosting (XGBoost). In this technique, the enhancement is done using deep autoencoder techniques for feature selection and dimensionality reduction.
- Developing Tuning rules of PSO to maintain convergence of optimal solutions and balance exploration and exploitation of the model hyperparameters.
- For multi-class LOS classification, the PSO-XnB model is evaluated on performance metrics, i.e., accuracy, F1 score, precision, recall, and area under the curve (AUC) score. Then, it is compared with other traditional ensemble models.

This study is structured as follows: Section 2 presents the related works on the importance of feature selection and classifiers for predictions. Section 3 elaborates on the proposed PSO-XnB framework, including the methodology and model design. Section 4 presents experimental observations and performance results comparing the PSO-XnB model with traditional ensemble methods. Finally, Section 5 concludes the findings and discusses potential future directions.

## 2 Related works

This section presents a literature survey highlighting previous research works limited to the past 4 to 5 years based on the importance of feature selection and the role of classifiers for predictions. This section will also present various feature selection methods used with classifiers to improve the medical diagnostic process. Shah et al. (2020) focused on improving the feature selection by combining the mean fisher-based feature selection algorithm (MFFSA) and accuracy-based feature selection algorithm (AFSA). The authors used a hybrid combination of feature selection methods to train and using principal component analysis (PCA), they selected the best features. These selected features were further trained with an RBF-based support vector machine (SVM); the overall accuracy scored was 92.1% using the Switzerland heart disease dataset on 123 instances. The Fisher score algorithm (FSA), F_score algorithm (FA), and extra trees classifier algorithms (ETCA) were used by Nasarian et al. (2020) to select features with the highest scores, specifically targeting the top 30%, 40%, and 50%. Subsequently, the selected features were trained with four baseline classifiers, namely, Decision Tree (DT), Gaussian Naive Bayes (GNB), Random Forest (RF), and XGBoost classifiers. The average accuracy scored from the four models was 83.94%, 81.58%, and 92.58%, using Hungarian, Long-beach-va, and Z-Alizadeh Sani datasets, respectively. Another work by Amarbayasgalan et al. (2021) used a two-step approach where PCA first divides initial data into commonly distributed and highly biased groups. In the next step, two variational autoencoders (VAE) were used to add samples in highly biased groups. Finally, highly biased group samples were trained with two Deep Neural Networks (DNN) models, and the model scored 89.2% accuracy and 91.5% F1 score. Mienye and Sun (2021) proposed a DL approach to improve heart disease prediction, utilizing a stacked sparse autoencoder network (SSAE) for feature selection. The selected features were trained with the soft-max classifier optimized by PSO; with this multilayer architecture, the authors scored 97.3% accuracy using the Framingham dataset. Another work used a sparse autoencoder (SAE) and softmax classifier; this combination scored 91% accuracy, as reported by Ebiaredoh-Mienye et al. (2020). This work did not use any optimization step; therefore, the model's parameters were not fully optimized for heart disease prediction. Ali et al. (2019) focused on eliminating irrelevant features followed by preprocessing with the statistical model chi-square (χ2). The over-fitting issue was minimized by configuring the χ2-DNN model through an exhaustive search strategy, because of which the model achieved 93.3% accuracy in heart disease prediction.

In discussing the use of ML models, Miriyala et al. (2021) used a single LGBM to diagnose CAD in 1,190 instances, reporting 93.3% accuracy; the model has an overfit issue, which can be solved through optimization. Barfungpa et al. (2023) optimized features using an enhanced sparrow search algorithm (E-SSA). Their focus was to minimize the dimensionality of data using deep-dense residual attention Aquila convolutional network (Deep-DenseAquilaNet). The model's approach was to update the weights using the Aquila optimization algorithm; this resulted in 99.57% accuracy using the comprehensive heart dataset with <2,000 instances. A heart disease prediction model employing a Chaos Game Optimization Recurrent Neural Network (CGO-RNN) model was reported by Alam and Muqeem (2023). The authors used integrated Kernel Principal Component Analysis (KPCA) as a strategic choice for handling the data's dimensionality and computational intensity. The model novelty helped the authors to achieve an accuracy of 98 ± 0.9%. However, the works reported by Barfungpa et al. (2023) and Alam and Muqeem (2023) could not converge during optimization.

As the research advanced, the LOS performance was improved using various ML models. In this context, Harerimana et al. (2021) propose three classes of LOS, where a hierarchical attention network (HAN) was trained with the MIMIC-III text-oriented dataset, achieving an AUC-ROC score of 0.82. Zou et al. (2023) used MIMIC-III and divided it into three datasets with different feature sizes, and the LOS labels from the datasets were extracted from the PostgreSQL DBMS. The authors proposed a generative adversarial network (GAN) called Wasserstein-GAN to predict the LOS class with a gradient penalty. For three datasets and various LOS ranges, the highest class scored 96.6% accuracy among other classes. Another work by Hempel et al. (2023) performed prediction using the MIMIC-IV dataset; in this work, the LOS was categorized into two classes: short and long stay. The authors used the default parameters of the model in optimization and classifier training using Logistic Regression (LR), RF, SVM, and XGBoost; the result shows that RF scored the highest accuracy of 81%. A framework for LOS prediction using RF with three over-sampling and three under-sampling techniques was proposed by Alsinglawi et al. (2022). The oversampling technique using RF scored 98% accuracy with a 95% confidence interval. González-Nóvoa et al. (2023) developed the XGBoost model optimized with Bayesian techniques to predict early ICU readmissions. The authors utilized SHAP to determine important features, scoring 0.92 ± 0.03 AUC-ROC.

In discussing LOS prediction using the MIMIC dataset for other diseases, El-Rashidy et al. (2022) developed a predictive model for detecting sepsis in patients admitted to the post-ICU during the initial 6 h of their stay. This innovative approach combined the strengths of the non-dominated sorting genetic algorithm-II (NSGA-II) and neural networks to extract optimal features. This hybrid model was followed by a classifier-stacked deep ensemble learning model to train the dataset. This model scored an accuracy of 91.3% and AUC score of 0.906. The effect of lung cancer on ICU patients was studied by Qian et al. (2023) using the MIMIC-III dataset. The study analyzed 1,242 ICU admissions, scaling them on illness severity AUC score; the predicted short-term and long-term mortality scores were 0.714 and 0.717, respectively. Bozkurt and Aşuroglu (2023) targeted their model in LOS prediction for patients admitted with breast, lung, prostate, and stomach cancer. The authors used the MIMIC-IV dataset and LR-based feature selection. The selected features were trained on five ML models: LR, DT, RF, SVM, and Multilayer Perceptron. The performance given by RF was the highest among others; the F1 scores ranged from 0.73 to 0.82, and AUC-ROC scores ranged from 0.88 to 0.96. The research discussed in this section 2 is compared in Table 1 according to the problem statement, the models used, and the limitations observed.

**Table 1:** Comprehensive literature survey table.

**References**	**Problem statement**	**Methods used**	**Limitations**
Shah et al. (2020)	Enhancing classifier performance by feature selection	MFFSA and AFSA with PCA and RBF-SVM	Sensitivity to parameter tuning
Nasarian et al. (2020)	Ranking features for medical diagnostics	FS, FA, ETCA with DT, GNB, RF, and XGBoost	Potentially high computational cost and complexity toward interpretations
Amarbayasgalan et al. (2021)	Impact of living factors identification using feature selection for CAD	PCA with VAE and DNNs	High computational intensity
Mienye and Sun (2021)	Feature selection-based CAD identification	SSAE with PSO and Softmax	Overfitting and high computational demands
Ebiaredoh-Mienye et al. (2020)	Heart disease diagnosis with feature reduction	SAE with Softmax	Potential for model overfitting and generalizability
Ali et al. (2019)	Enhancing feature quality for cardiac disease diagnosis	χ2 with Optimally Configured DNN	Underfitting and the inherent complexity of tuning deep neural networks
Miriyala et al. (2021)	Diagnosis of CAD	Single LightGBM	Overfitting, need for optimization
Barfungpa et al. (2023)	Feature selection and optimization for heart disease prediction	E-SSA with Deep-DenseAquilaNet	Vulnerability to convergence
Alam and Muqeem (2023)	Heart disease prediction with optimization	CGO-based RNN with KPCA	Vulnerability to convergence
Harerimana et al. (2021)	Predicting LOS with HAN	HAN with SMOTE	Overfitting in imbalanced data
Zou et al. (2023)	Realistic LOS distribution using GAN	WGAN with gradient penalty	Misleading predictions in unseen scenarios could be a limitation.
Hempel et al. (2023)	LOS prediction on ICU patients	LR, RF, SVM, XGBoost	Computational intensity
Alsinglawi et al. (2022)	LOS prediction framework	RF over-sampling and under-sampling techniques	Data biases, overfitting due to oversampling techniques
González-Nóvoa et al. (2023)	Early ICU readmission prediction	XGBoost with Bayesian, SHAP for feature importance	Computational intensity and interpretability
El-Rashidy et al. (2022)	Sepsis prediction in ICU	NSGA-II with neural networks and stacked deep ensemble learning	Model transparency and explainability issues
Qian et al. (2023)	Short-term mortality prediction for Lung patients in ICU	Multivariate LR	Potential applicability issue due to low instances
Bozkurt and Aşuroglu (2023)	Mortality prediction in cancer patients	LR, DT, RF, SVM, MLP	Data imbalance and noise were observed

The recent pandemic has forced researchers to use nature-inspired metaheuristic optimization algorithms to target complex datasets. Among these, algorithms such as the capuchin search algorithm (Braik et al., 2023a), particle swarm optimization sine cosine algorithm (Somgiat and Chansamorn, 2022), whale optimizer (Nadimi-Shahraki et al., 2022), and snake optimizer (Braik et al., 2023b) have shown promising results in ML and DL applications. However, our study uses PSO optimization due to its robustness in determining complex, multidimensional search spaces from clinical data and its convergence toward optimal solutions (Kennedy and Eberhart, 1995). The existing works primarily focused on the binary classification of the LOS. In contrast, our proposed model PSO-XnB focuses on showing the best performance across multiple categories where the model is implemented with deep autoencoders for feature selection and dimensionality reduction, XGBoost for predictive modeling, and PSO for fine-tuning the model's hyperparameters. With the development of specific tuning rules, our model also achieves a balance between exploration and exploitation, leading to enhanced model convergence and accuracy. Section 3 discusses the strategic choices for developing PSO-XnB in detail.

## 3 Materials and methods

**Figure 1:**
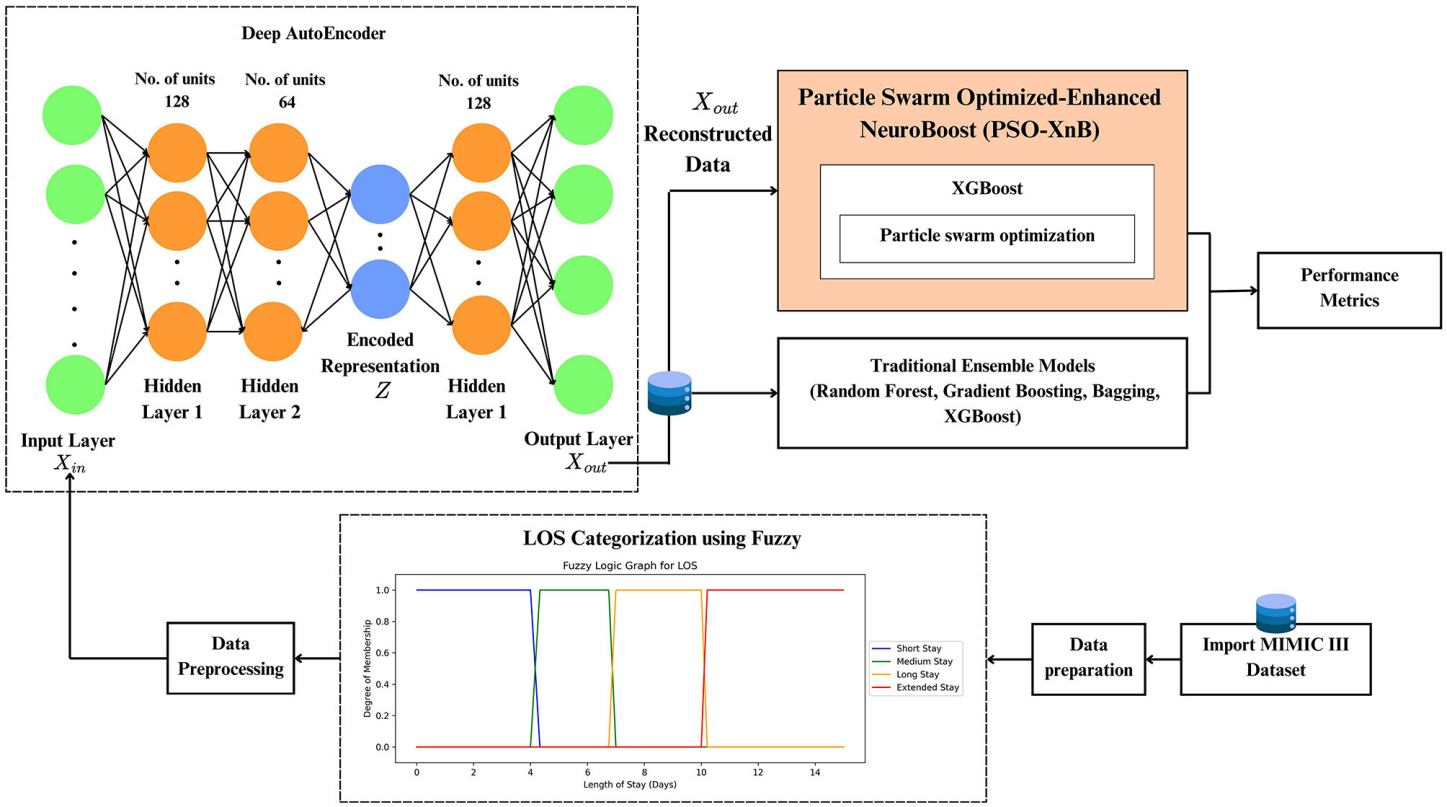
The proposed framework for the PSO-XnB model.

Our methodology of the PSO-XnB framework is depicted in Figure 1. The work starts with data preparation, followed by preprocessing, discussed in Section 3.1.

The framework of PSO-XnB model development to predict LOS for CAD patients is discussed in Section 3.2.

### 3.1 Dataset preparation and preprocessing

This study uses the MIMIC-III clinical dataset (Goldberger et al., 2000; Johnson et al., 2016). From the large dataset, our selection process exclusively focuses on medical cases relevant to CAD disease. Therefore, the keyword extraction method is performed from the “admit diagnosis” feature. Keywords such as CAD, heart, coronary, chest pain, myocardial infarction, angina, cardiac aortic, and STEMI were utilized for extraction. After this extraction, our final dataset included 9,989 patient instances with 23 features. These features cover admission details, clinical and care metrics, and medical outcomes, including diagnoses, procedures, inputs/outputs, and length of stay. The continuous patient records were gathered from the dataset. Data handling practices were ensured to maintain the study's integrity, reliability, and reproducibility. The “LOSdays” (length of stay in days) is selected as a target variable to predict hospital stay durations. This work categorizes the LOS classes with fuzzy followed by three steps. In the first step, the fuzzy sets for each LOS category are defined as short, medium, long, and extended stays. The second step is to define fuzzy rules based on data summary statistical analysis to understand the inherent variability and patterns. The rules given are as follows (Jena et al., 2022):

- **IF** LOSDays are ≤ 4.33 days, **THEN** the stay is likely a “short stay.”
- **IF** LOSDays are >4.33 days AND are ≤ 6.75 days, **THEN** the stay is likely “medium stay.”
- **IF** LOSDays are >6.75 days AND are ≤ 10.21 days, **THEN** the stay is likely a “long stay.”
- **IF** LOSDays are >10.21 days, **THEN** the stay is likely an “extended stay.”

**Figure 2:**
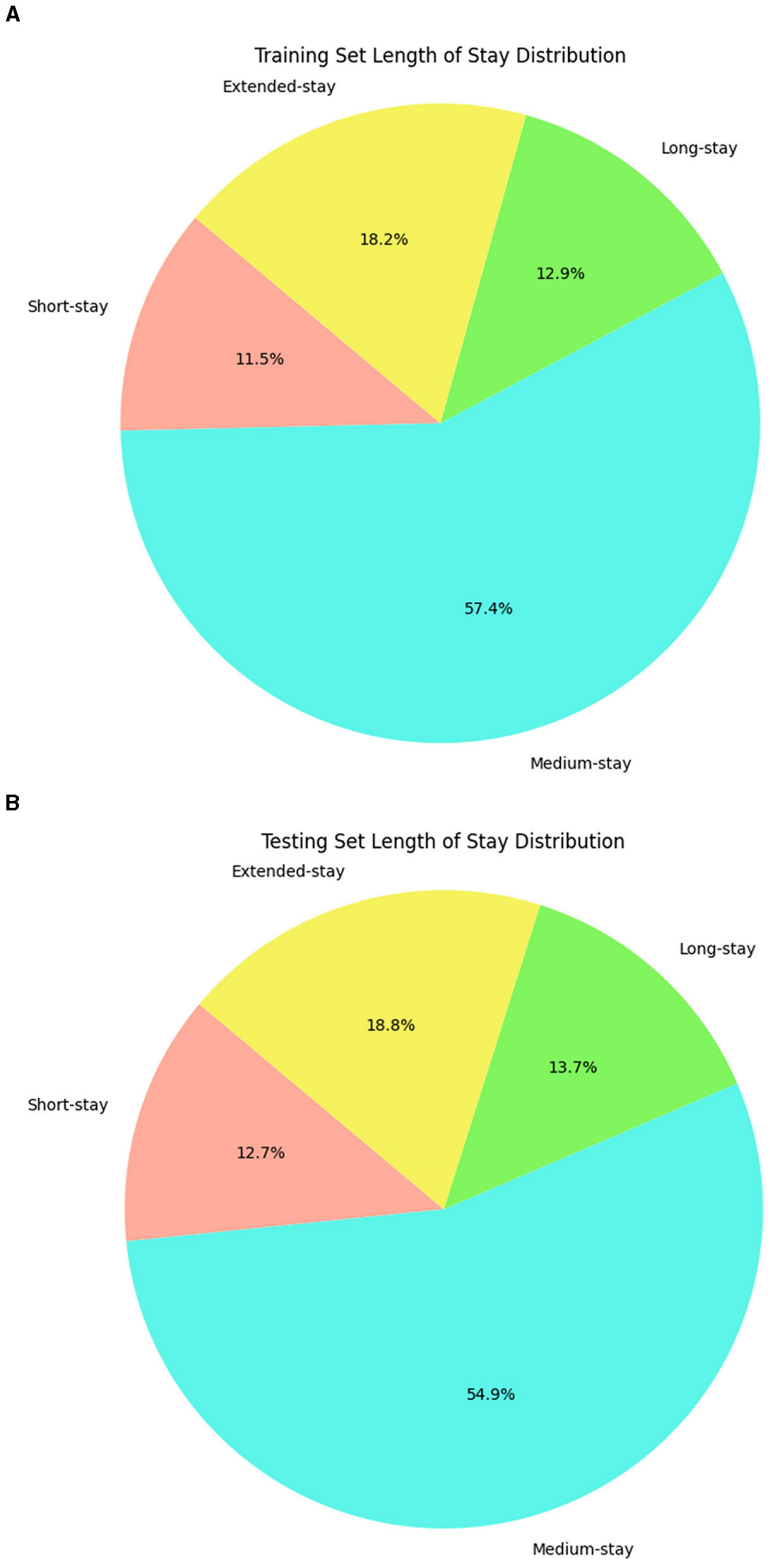
Distribution of LOS on train test splits. **(A)** Training dataset. **(B)** Testing dataset.

This observation defines fuzzy sets with boundaries reflecting the data's natural distribution, not arbitrary intervals. These rules can capture overlaps and gradations of the LOS class. In the final step through the inference mechanism, fuzzy rules are applied with the trapezoidal membership to identify the complex patterns where a patient's LOS can simultaneously exhibit characteristics of multiple categories. These rules help to make predictions more accurate and adaptable because they are considered with different possibilities and variations in LOS. Further, label encoding was used as a preprocessing step to process the categorical variables into numerical format (Boulif et al., 2023), and this preprocess data was split into training and testing sets. The LOS classes obtained from a fuzzy set of rules are distributed across training and testing data, which is visualized in Figure 2, revealing the data imbalance. Therefore, the Synthetic Minority Over Sampling Technique (SMOTE), a standard technique in ML, is applied to balance the data. This technique effectively addresses the imbalance issue and augments the training and testing datasets from 7,991 and 1,998 instances to 18,188 and 4,548 instances. The data augmentation does not fabricate new data randomly. Instead, it analyses the existing data to identify underlying patterns specifically within the underrepresented classes and creates additional synthetic data to enhance those minority class representations.

### 3.2 Proposed PSO-XnB model

After data preprocessing, the MIMIC III dataset shows high dimensionality due to the multiplicity of features and instances. Therefore, our work uses a deep autoencoder, an artificial neural network developed with TensorFlow (Abadi et al., 2016) and Keras (Chollet, 2015). The deep autoencoder can find patterns within the data that are not easily visible due to the complex relation*X*ships between features in medical datasets (Bank et al., 2023; Shinde et al., 2023). Let us denote the original data with the dimensions of *m*×*n*, where *m* and *n*, denote the number of instances and features. The autoencoder is a combination of encoder and decoder, and it is initialized with an encoder function *E*(*X*, θ_*E*_), where θ_*E*_ determines the network parameters of the encoder *E*. The encoder network starts with the input layer of the original data *X*_*in*_ that matches the feature space of the entire data where $X_{in}={\mathbb{R}}^{n}$, and ℝ^*n*^ determines the n-dimensional space of real numbers. In this encoder, the two mappings are typically used to reduce the dimensionality of the input data. The first and second mappings *E*_1_ and *E*_2_ are used as functions to transform data from ℝ^*n*^ → ℝ^128^ and ℝ^128^ → ℝ^64^. The function *E*_1_ transforms input data from n-dimensions to 128 dimensions by adding features and *E*_2_ then reduces this data from 128 dimensions to 64 dimensions, focusing on obtaining the most relevant features. Thus, and use the rectified linear activation (ReLU) function to introduce non-linearity forming latent encoded representation *Z* = *E*_2_(ReLU(*E*_1_(*X*_*in*_))).

The subsequent decoder function *R*, mirrors the two encoder mappings as $R_{1}:{\mathbb{R}}^{64}\to {\mathbb{R}}^{128}$, and $R_{2}:{\mathbb{R}}^{128}\to {\mathbb{R}}^{n}$. These mappings used in the decoder transform the encoded low-dimensional data back to the reconstructed original data, so the output of the decoder is *X*_*out*_ = *R*_2_(ReLU(*R*_1_(*Z*))). The complete operation of the autoencoder *A* functions as *A*(*X*; θ_*E*_) = *R*(*E*(*X*)), where *X*, parameterized by θ_*E*_, determines the process initially transformed by the encoder *E* into lower dimensional representations, and then the decoder reconstructs the input data from this encoder representation. The optimization of encoder parameters θ_*E*_ continues iterations with the mean square error until the lowest possible difference between the original data and the reconstructed data is achieved. The reconstruction data obtained from the autoencoder at minimal loss shows reduced dimensionality and a focus on important features.

**Figure 3:**
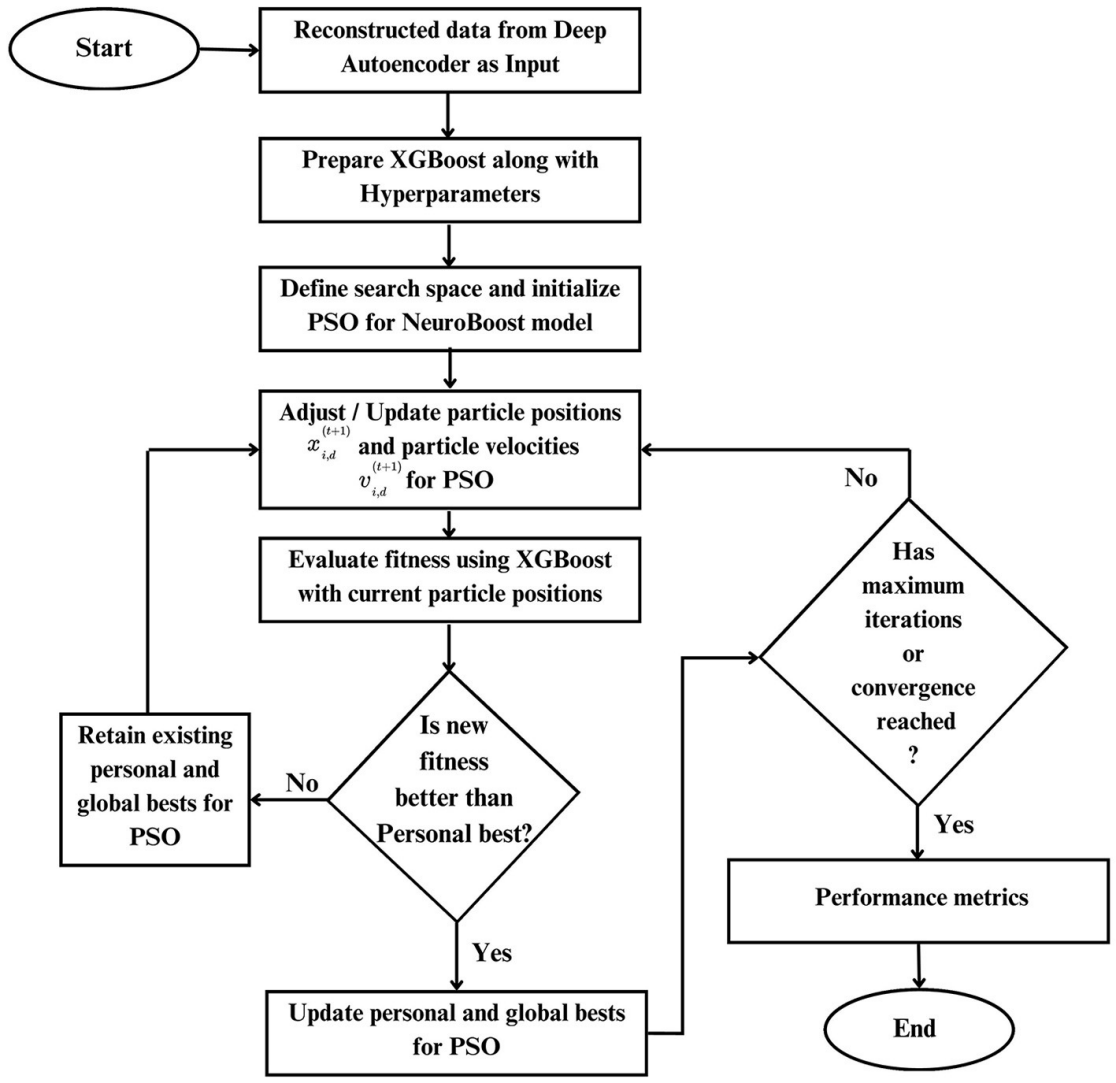
Flowchart of PSO-XnB model.

This proposed approach uses the reconstructed data as input for the XGBoost classifier to be optimized with PSO (Farahnakian and Heikkonen, 2018). Le et al. (2019) and Jiang et al. (2020) previously developed the PSO-XGBoost model, but their studies faced challenges in predicting minority classes (Jiang et al., 2020) and handling a high number of input variables (Le et al., 2019). In contrast, our model, PSO-XnB, addresses these issues using autoencoders, an effective approach to predict outcomes across all LOS classes. The flowchart of the PSO-XnB model is shown in Figure 3. Our work uses the XGBoost model, enhanced by a gradient-boosting technique, for efficient performance in real-world applications (Chen and Guestrin, 2016). The model XGBoost trained without regularization could overfit the training data, capturing noise from the underlying patterns (Kigo et al., 2023). Therefore, fine-tuning the hyperparameters is crucial to balance the model's performance, ensuring it generalizes well without overfitting (Yewale et al., 2023). In this study, Particle Swarm Optimization (PSO) is used for hyperparameter tuning, a method inspired by natural patterns in bird flocking and fish schooling introduced by Kennedy and Eberhart (1995). This optimization, combined with deep autoencoder reconstructed data, aims to improve the predictive capacity of the XGBoost model, yielding a highly accurate outcome. The entire model development is structured into seven distinct steps:

1. *Encoding Train and Test Input Data:*

The reconstructed data *X*_*out*_ and the target variable *Y* is divided into training and testing sets defined in Equation (1):


(1) $$\begin{array}{l} \left(X_{out,train},Y_{train}\right)\left(X_{out,test},Y_{test}\right)=\text{split}\left(X_{out},Y\right) \end{array}$$

Where *X*_*out, train*_, *Y*_*train*_ and *X*_*out, test*_, *Y*_*test*_ represents training and testing input and output data.

2. *Defining Hyperparameters for XGBoost:*

The hyperparameters of the XGBoost model utilized in this study are represented by a vector, θ_*X*_ = [*lr, ne, md, mcw, ss, cbt*], and the variables in the vector are detailed in Table 2.

**Table 2:** Shows the configured information of XGBoost Hyperparameters.

**Hyper-parameter**	**Default value**	**Range**	**Explanation**
learning_rate (lr)	0.1	0.01–0.3	Sets the minimal loss function and controls the step size for each iteration.
n_estimators (ne)	100	50–1,000	The number of trees inside the group. Better speed, but more computation, is possible with more trees
max_depth (md)	3	3–10	Maximum depth to which a tree can grow. The model's complexity and the likelihood of overfitting are both affected by this parameter.
min_child_ weight (mcw)	1	1–10	Maximum depth to which a tree can grow. The model's complexity and the likelihood of overfitting are both affected by this parameter.
subsample (ss)	1	0.5–1.0	Subsample ratio of the training instances. Although a lower number avoids overfitting, underfitting might occur with values that are too low.
colsample_bytree (cbt)	1	0.5–1.0	When building each tree, use the subsample ratio of columns. Each created tree undergoes subsampling once.

3. *Define Search Space and PSO Initialization:*

Initially, search space is defined for PSO as a multidimensional space, aiming to optimize different hyperparameters of the XGBoost model within each dimension. The range for each XGBoost hyperparameter used in this process determines the boundaries of the search space (El Dor et al., 2012). Before starting the PSO process, the configuration parameters and their values play an important role in directing the particle's movement throughout the hyperparameter space. Our configurations start with ten particles, each representing a possible solution within the XGBoost hyperparameter search space. This specific number of particles is selected to maintain computational efficiency while providing a diverse set of initial candidate solutions. Six dimensions are categorized for each particle. The hyperparameters of XGBoost are outlined in Table 2. For the learning factors, the local learning factor (C1) and global learning factor (C2) are set at 0.5 and 0.3. The setting value of C1 guides each particle toward its best optimal position by fine-tuning solutions based on past performance. The lower C2 value, on the other hand, encourages particles to seek out new potential solutions in new regions of the search space. Our model using PSO is limited to 50 iterations because this value achieved efficient convergence at initial tests. Finally, ten independent runs were conducted to ensure the reliability of the optimization process. These runs help to validate the stability and replicability of our hyperparameter tuning results. After preliminary testing, the best hyperparameters were ultimately chosen based on observing the optimal balance between computational efficiency and the depth of exploration within the hyperparameter space, ensuring the robustness of our findings.

Each particle's *i* initial position and velocity in PSO are described by vectors and ${v_{i}}^{\left(0\right)}$ that correspond to the values of hyperparameters, i.e., learning rate, number of estimators, max depth, min child weight, subsample size, and colsample by tree. The position vector represents the initial point in the search space, which is defined as ${x_{i}}^{\left(0\right)}=\left[lr_{i},ne_{i},md_{i},mcw_{i},ss_{i},cbt_{i}\right]$. The velocity vector sets the initial speed as zero or a value close to zero, allowing particles to explore the search space without bias, defined as${v_{i}}^{\left(0\right)}=\left[{v_{lr}}_{{\;}_{i}},{v_{ne}}_{{\;}_{i}},{v_{md}}_{{\;}_{i}},{v_{mcw}}_{{\;}_{i}},{v_{ss}}_{{\;}_{i}},{v_{cbt}}_{{\;}_{i}}\right]$.

4. *PSO Iterative Process:*

In the iteration process *t*, the particle positions and velocities are updated using the predefined PSO rules. Therefore, the velocity for each dimension *d* of *i* is adjusted and updated by Equation (2):


(2) $$\begin{array}{l} v_{i,d}^{\left(t+1\right)}=w\;\cdot \;v_{i,d}{\;}^{\left(t\right)}+c_{1}\cdot \;r_{1}\;\cdot \;(p_{i,d}{\;}^{best}-x_{i,d}{\;}^{\left(t\right)})\\ \;+\;c_{2}\cdot r_{2}\cdot (g_{d}{\;}^{best}-x_{i,d}{\;}^{\left(t\right)}) \end{array}$$

From the above equation, *w* determines the inertia weight that balances exploration and exploitation, impacting the particle's current velocity, *c*_1_ determines cognitive coefficients, and *c*_2_ determines social acceleration coefficients. *r*_1_ and *r*_2_ are random numbers ranging between 0 to 1 providing stochasticity to the search, ${p_{i,d}}^{best}$ which is the best individual position found in the *d* and ${g_{d}}^{best}$ is the best position obtained in the swarm by any particle found in *d*. The term $c_{1}\cdot r_{1}\cdot \left({p_{i,d}}^{best}-{x_{i,d}}^{\left(t\right)}\right)$ represents a particle's memory of its best position, marked as a cognitive component, and $c_{2}\cdot r_{2}\cdot \left({g_{d}}^{best}-{x_{i,d}}^{\left(t\right)}\right)$ represents the particle's cooperation with the swarm as a social component. Therefore, with the iterations, each particle's position ${x_{i,d}}^{\left(t\right)}$ in the search space updates according to the velocity ${v_{i,d}}^{\left(t+1\right)}$. This updated position becomes the candidate solution for the next iteration ${x_{i,d}}^{\left(t+1\right)}$, defined in Equation (3).


(3) $$\begin{array}{l} {x_{i,d}}^{\left(t+1\right)}={x_{i,d}}^{\left(t\right)}+{v_{i,d}}^{\left(t+1\right)} \end{array}$$

Through these equations, the PSO algorithm allows each particle to explore the search space, adjusting its trajectory based on its own experience and the swarm's collective knowledge. By updating each particle, the swarm converges to the best solution of hyperparameters that results in the best model performance according to the chosen fitness metric.

5. *Fitness Evaluation and Model Training with Dimensionality:*

The XGBoost model is trained using the hyperparameters represented by the particle's position (Qin et al., 2021), and the F1 score is chosen as the fitness function because it balances precision and recall and can predict all classes accurately. This fitness metric is especially suitable for imbalanced datasets. The fitness function used in this work is defined by Equation (4) as:


(4) $$f(x_{i})=\text{F1 ​​}\_\text{​​ Score(XGBoost}(X_{out},y,{\left\{x_{i,d}\right\}}_{d=1}^{D}))$$

The Equation (4) represents *f*(*x*_*i*_) a fitness function of the particle *i*, and calculates the F1 score of the XGBoost model trained with the reconstructed data and target, using the hyperparameters of position vectors ${\left\{x_{i,d}\right\}}_{d=1}^{D}$ across all dimensions *D* for the particle *i*. However, depending on the specific requirements of the task, other metrics such as accuracy or precision could also serve as the fitness score.

6. *PSO Convergence to Best Hyperparameters with Dimensionality:*

To obtain the best hyperparameters $\theta_{X}^{best}$ across all dimensions *D*, the global best positions defined across each dimension are defined in Equation (5) as:


(5) $$\begin{array}{l} \theta_{X}^{best}={\left\{{g_{d}}^{best}\right\}}_{d=1}^{D} \end{array}$$

The PSO algorithm fine-tune the hyperparameters iteratively to minimize a convex loss function. Simultaneously, the F1 score is also used as a fitness measure. This dual-focus strategy ensures the best hyperparameter solutions $\theta_{X}^{best}$, which enhances the model's accuracy and reliability. To ensure the reach of the convergence of the PSO, 10 independent runs with 50 iterations are executed. The optimal set of hyperparameters is then selected based on the lowest convex loss observed from the XGBoost model evaluations during these runs, as presented in Table 3.

**Table 3:** Best XGBoost Hyperparameters obtained from PSO for ten independent runs.

**Ind. run**	**Learning rate**	**N_estimators**	**Max depth**	**Min child weight**	**Subsample**	**Colsample bytree**	**Best loss**
1	0.1286	102	9	5.0232	0.9578	0.5423	0.0128
2	0.2611	66	9	5.3988	0.8418	0.5094	0.0129
3	0.2384	180	9	2.8733	0.8085	0.5831	0.0128
4	0.2478	109	9	4.676	0.814	0.5362	0.0129
5	0.2236	159	8	3.8692	0.9969	0.5470	0.0128
6	0.1619	146	7	1.2177	0.9935	0.6768	0.0123
7	0.2936	161	9	3.6239	0.9968	0.5270	0.0129
8	0.2765	112	8	6.0241	0.9502	0.5149	0.0126
9	0.19	127	5	5.4939	0.8721	0.5387	0.0130
10	0.2127	112	10	2.0516	0.8496	0.7971	0.0125

7. *Training and Prediction with the Enhanced NeuroBoost Model with Dimensionality:*

Finally, the NeuroBoost model is trained with $\theta_{X}^{best}$, obtained from the PSO process defined by Equation (6) as:


(6) $$\begin{array}{l} Model_{NeuroBoost}^{Final}=Train\left(X_{out},y;\theta_{X}^{best}\right) \end{array}$$

The final model is expected to demonstrate enhanced performance due to being fine-tuned with the dimensionally optimized hyperparameters. Further, the model is employed across various classes of LOS as *C* = {*c*_*short*_, *c*_*medium*_, *c*_*long*_, *c*_*extended*_}, where each class in the set belongs to a specific LOS class. The model predictions on the reconstructed test data are defined by the Equation (7) as:


(7) $$\begin{array}{l} {\hat{y}}_{test,k}=Model_{NeuroBoost}^{Final}{\left(X_{out,test}\right)}_{k} \end{array}$$

Therefore, the final objective function of the PSO-XnB model, $Ob{j_{\theta_{X}^{best}}}^{\left(t\right)}$ at iterations *t*, sums over all classes *k*, and computes with the best parameters given by Equation (8)as:


(8) $$\begin{array}{l} Ob{j_{\theta_{X}^{best}}}^{\left(t\right)}=\overset{n}{\underset{m=1}{\sum }}\left[\underset{k\in C}{\sum }l\left({\hat{y}}_{m,k}^{\left(t-1\right)},y_{m,k}\right)+{\hat{f}}_{\theta_{X}^{best}}^{{\;}^{\left(t\right)}}{\left(x_{m}\right)}_{k}\right]+\Omega \left({\hat{f}}_{\theta_{X}^{best}}\right) \end{array}$$

Where *l* is a differentiable convex loss function that measures the difference between actual *y*_*m, k*_ and predicted test ${\hat{y}}_{m,k}^{\left(t-1\right)}$ observations of instances *m* with *k*, $\Omega \left({\hat{f}}_{\theta_{X}^{best}}\right)$ is the regularization term that penalizes the complexity of the model to prevent overfitting. ${\hat{f}}_{\theta_{X}^{best}}^{{\;}^{\left(t\right)}}{\left(x_{m}\right)}_{k}$ is the predictive function of the model using the best hyperparameters for input features of instances *x*_*m*_ across all target classes *k*. Due to the internal mechanism of XGBoost, the L1 regularization terms get explicitly modified as best based on cost per leaf γ_*best*_ and the L2 regularization term based on leaf weights λ_*best*_, both optimized via hyperparameters defined in the Equation (9):


(9) $$\begin{array}{l} \Omega \left({\hat{f}}_{\theta_{X}^{best}}\right)\;=\;\gamma_{best}T\;+\;\frac{1}{2}\lambda_{best}\;{||\hat{W}||}^{2} \end{array}$$

Here, *T* is the number of leaves in the tree, and $\hat{W}$ is the vector of scores on the leaves. Finally, the performance metrics are very important to evaluate the performance of a multi-class classification problem, as discussed in Miriyala et al. (2021). In this work, the overall model's performance is observed using accuracy as a primary metric, and the F1 score acts as an alternative metric to understand the positive class predictions from precision and recall across each LOS class. The Precision-Recall (PR) curve and Receiver Operating Characteristic (ROC) curve are also examined to gain insights into the model distinguishing the target classes. These visual representations offer an understanding of its capabilities. The Average Precision (AP) is also derived from the PR curve and effectively summarizes the model's performance for each category (Le et al., 2019).

## 4 Experimental results and performance observations

**Figure 4:**
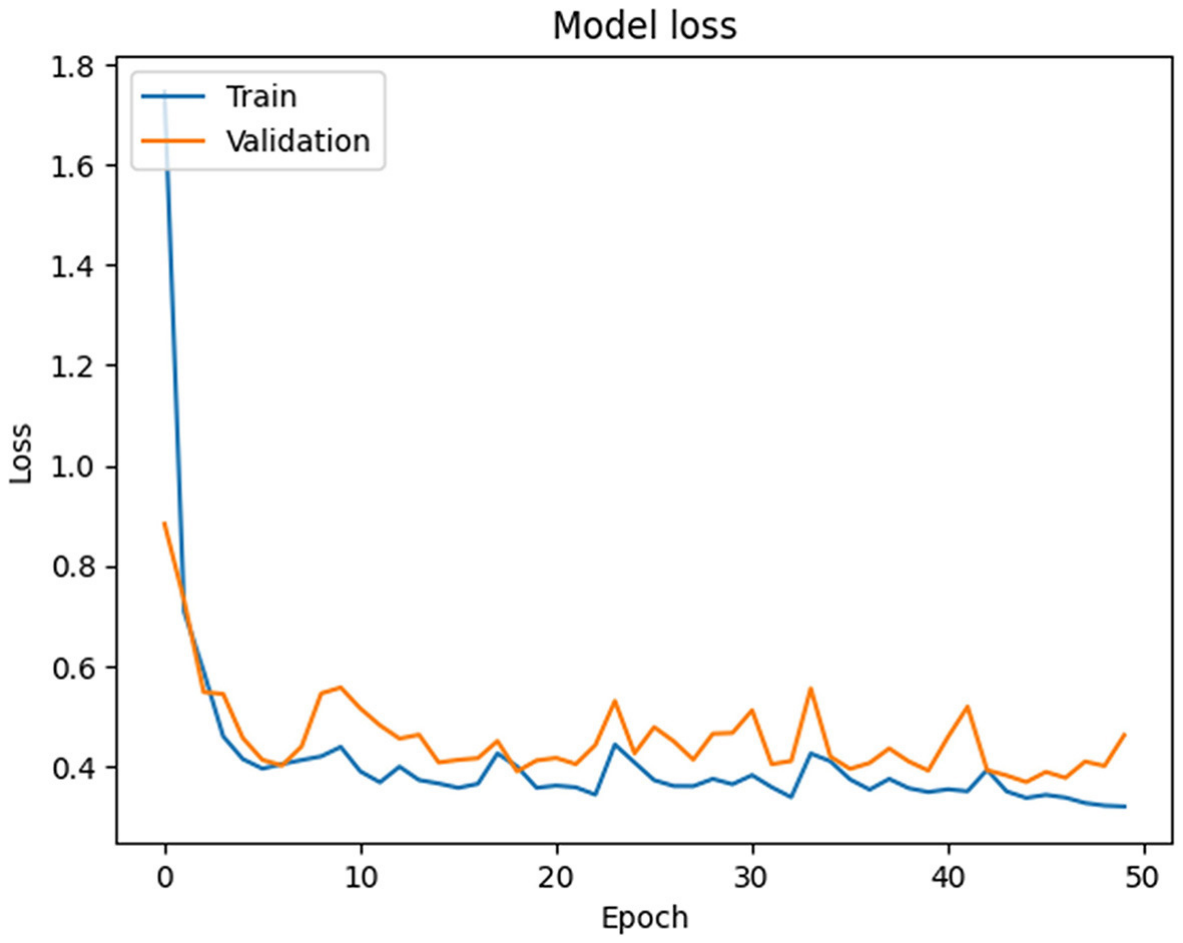
Deep autoencoder epoch-wise training and validation loss.

In this section, the simulation setup of the PSO-XnB model is performed on a computational environment with NVIDIA^®^ GeForce RTX™ 3050 Ti graphics card, Windows operating system, and an 11th generation Intel Core™ i7-11390H CPU for simulation. PyCharm is the simulation environment used with the Python 3.6.3 programming language (Van Rossum and Drake Jr, 1995). The libraries that were utilized were NumPy, Pandas, Matplotlib, SciPy, scikit-learn (Fandango, 2017), PySwarms (James and Miranda, 2018), Bayesian optimization (Nogueira, 2014), imbalanced-learn (LemaÃŽtre et al., 2017), TensorFlow (Abadi et al., 2016), SHAP (Lundberg and Lee, 2017), and a few other utilities for preprocessing data and evaluating models from scikit-learn. Our primary data source was the MIMIC III dataset, and the Pandas library was used mostly in data preparation. Initially, the reconstructed data is obtained from deep autoencoder data with the minimum MSE loss shown in Figure 4, where the training and validation loss starts at 1.7450 and 0.8830 at Epoch 1. However, over 50 iterations, the training loss consistently reduced to 0.3567, and the fluctuations in the validation loss were identified in Epochs 30 and 50, indicating the challenges in generalization. Therefore, to improve the model's performance toward the data, for further optimization is required.

### 4.1 PSO-XnB model results

**Figure 5:**
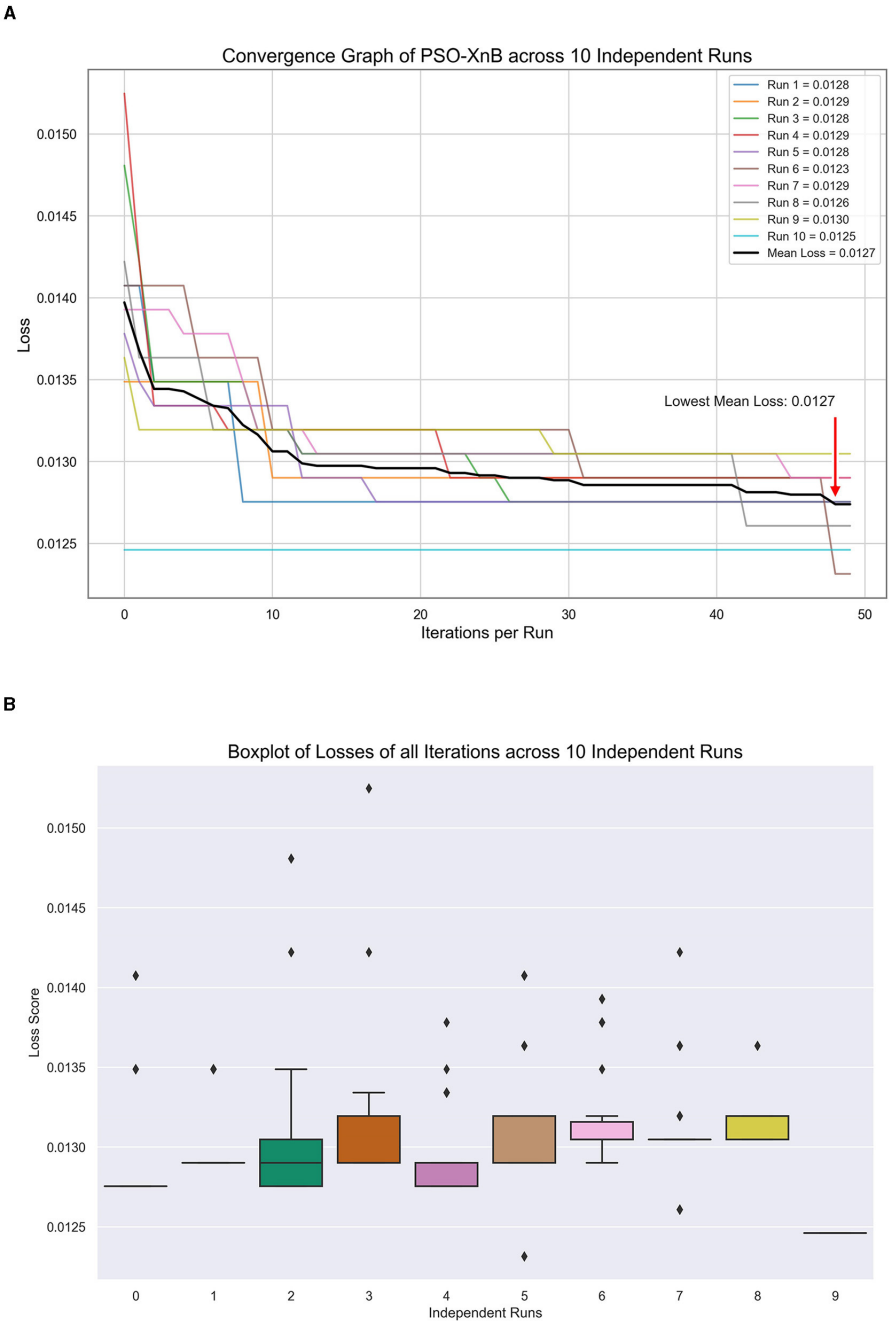
Model tuning results of PSO-XnB for ten independent runs. **(A)** Convergence graph. **(B)** Loss dispersion graph.

In our study, to predict the LOS, the best XGBoost hyperparameters from PSO are obtained to minimize convex loss, showing the characteristic exploratory nature of PSO. These values across ten independent runs are tabulated in Table 3. Each row corresponds to a separate optimization run, showcasing the unique set of hyperparameters. Figure 5A visualizes the PSO algorithm's efficacy, showcasing the convergence of loss for ten independent runs to a mean of 0.01275. Figure 5B, showing a boxplot, highlights a median loss of 0.0128, indicating a robust optimization process with narrow variability. From Table 3, the best and worst losses are observed as 0.013 and 0.0123, respectively, indicating tight clustering of outcomes and suggesting stability in the optimization process across ten independent runs. The standard deviation is 0.000217, and there is an extremely low variance. This visual and statistical confirmation of the PSO's performance underscores the reliability of our model for healthcare predictive analytics.

With the selected hyperparameters highlighted in Table 3, the PSO-XnB is trained on the test dataset. The performance metrics of PSO-XnB are calculated and shown in Table 4. The PSO-XnB on extended stay class scored high in precision, recall, and F1 scores metrics, suggesting that the model predicted accurately. However, the perfect recall score for extended stay could indicate a need for more diversity in the training set, potentially limiting the model's ability to handle a range of cases. On the other hand, there is a lower recall score for the medium stay class, indicating that there may be fewer true positive predictions for this category, which could be an area focusing on improving the model. The overall accuracy of the PSO-XnB was 98.8%, proving successful hyperparameter tuning using PSO-XnB. From these performance results, it is clear and promising that the deep autoencoder reconstructed data showed a considerable scope for future research aiming for refinement with the tools for broader applications in healthcare analytics.

**Table 4:** Confusion matrix scores of proposed PSO-XnB model.

**Class**	**Precision**	**Recall**	**F1 score**
Short stay	0.9795	0.9959	0.9876
Medium stay	0.9894	0.9620	0.9785
Long stay	0.9845	0.9931	0.9888
Extended stay	0.9988	1.0000	0.9994

### 4.2 Comparison with other models

In this subsection, the proposed model PSO-XnB is compared with other ensemble models such as Random Forest (RF) (Hempel et al., 2023), Gradient Boosting (GB) (Naemi et al., 2021), Bagging (Tully et al., 2023), and XGBoost (Hempel et al., 2023), to observe the effectiveness of predicting LOS.

**Figure 6:**
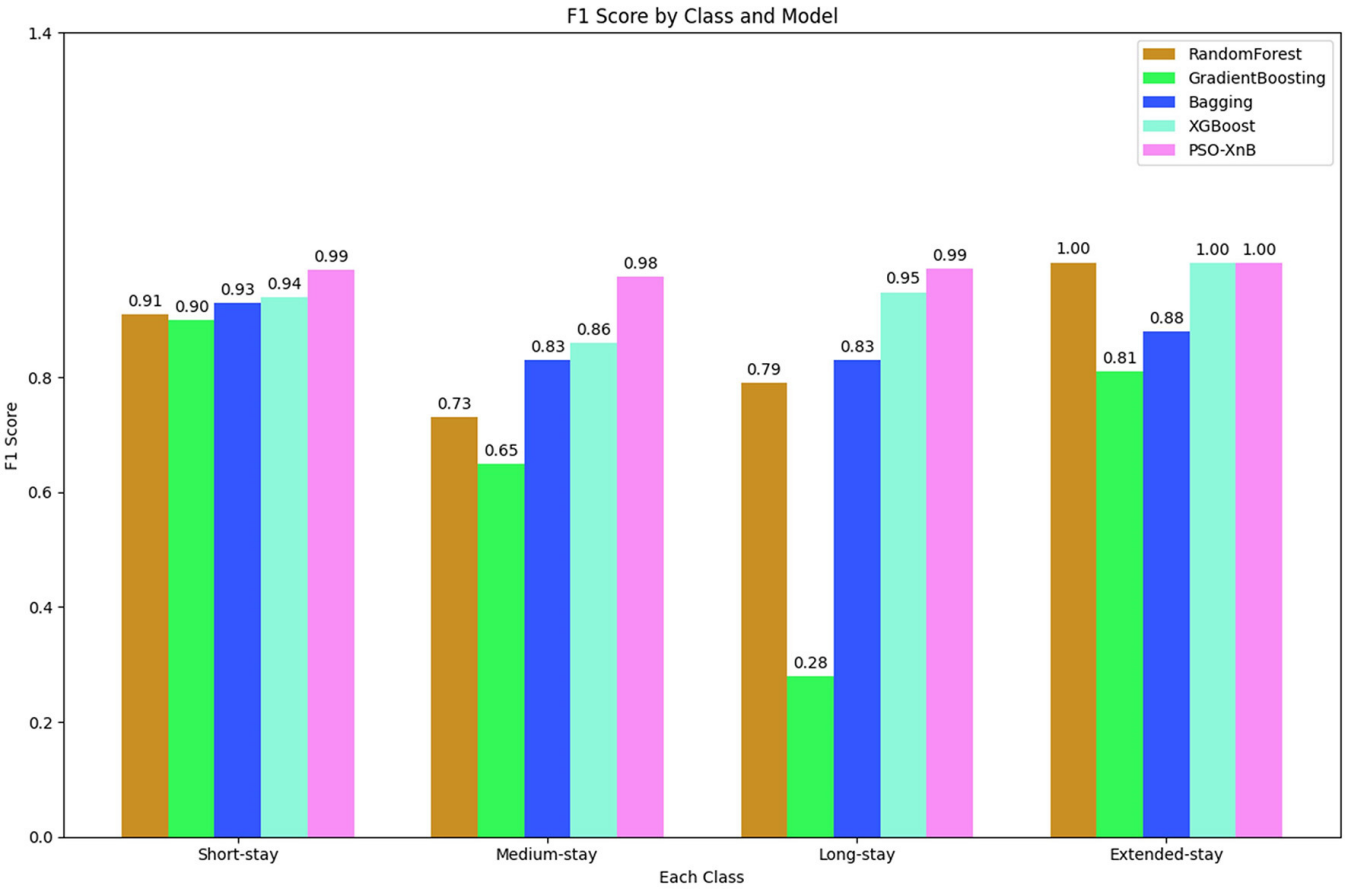
Comparison of F1 score of PSO-XnB across various models.

The overall accuracy of the other traditional ensemble models, such as RF, GB, Bagging, and XGBoost, scored 85.74%, 69.04%, 86.98%, and 94%. The F1 scores obtained from each ensemble model are compared with PSO-XnB, as shown in Figure 6, and the comparison of performance metrics is shown in Table 5. PSO-XnB showed the highest F1 scores of 0.99 for short stays, 0.98 for medium stays, 0.99 for long stays, and 1.00 for extended stays. The XGBoost scored second highest for three classes, i.e., short stay, medium stay, and long stay, and the RF model performed highest only for extended stay. Compared to the F1 score, gradient-boosting scores are the lowest in all four classes. Along with the F1 score, the precision and recall scores are also shown in Table 5, where the PSO-XnB model scored higher for each LOS class than other ensemble models.

**Table 5:** Comparison of performance metrics with proposed model and other ensemble models.

**Class**	**Model**	**Precision**	**Recall**	**F1 score**
	RF	0.92	0.90	0.91
	GB	0.96	0.85	0.90
SS	Bagging	0.89	0.97	0.93
	XGBoost	0.88	0.99	0.94
	**PSO-XnB**	**0.97**	**0.99**	**0.99**
	RF	0.69	0.78	0.73
	GB	0.51	0.91	0.65
MS	Bagging	0.83	0.83	0.83
	XGBoost	0.99	0.755	0.86
	**PSO-XnB**	**0.99**	**0.96**	**0.98**
	RF	0.83	0.75	0.79
	GB	0.6	0.18	0.29
LS	Bagging	0.83	0.84	0.83
	XGBoost	0.90	0.99	0.95
	**PSO-XnB**	**0.98**	**0.99**	**0.99**
	RF	0.99	0.99	1.00
	GB	0.79	0.83	0.81
ES	Bagging	0.92	0.84	0.88
	XGBoost	0.99	1.00	1.00
	**PSO-XnB**	**0.99**	**1.00**	**1.00**

The bold values for the PSO-XnB model represent the highest performance metric scores achieved in comparison to the other ensemble models for each respective class: SS, Short Stay; MS, Medium Stay, LS, Long Stay, and ES, Extended Stay.

For observing the significant validation, the consistent performance of PSO-XnB over the baseline ensemble models is performed with ten test splits, as shown in Supplementary Table S1. These evaluations are further statistically tested with the paired *t*-test to show the significance of the four LOS classes, i.e., short stay (SS), medium stay (MS), long stay (LS), and extended stay (ES). The results from the statistical analysis are summarized in Table 6. As reported in Table 6, the PSO-XnB model shows significance over ensemble models across all categories of LOS. For each class of short, medium, and long stay, the *p*-values are <0.001, showing that our model PSO-XnB is statistically significant compared to other baseline traditional ensemble models. A similar trend of *p*-values close to 0.001 is observed for an extended LOS stay. These lowest *p*-values determine a high degree of statistical confidence. Observing the statistical analysis, the PSO-XnB model demonstrates superiority in predicting the LOS categories compared to other baseline ensemble models.

**Table 6:** Statistical significance summary: PSO-XnB with other ensemble models on LOS classes.

**Descriptives**	**N**	**Mean**	**Median**	**SD**	**SE**	**Statistic**	**df**	***p***
PSO-XnB–SS	10	0.988	0.988	0.00254	8.04E-04	N/A	N/A	N/A
PSO-XnB–RF_SS	10	0.907	0.915	0.02457	0.00777	10.35	9	<0.001
PSO-XnB–GB_SS	10	0.898	0.9	0.02325	0.00735	11.9	9	<0.001
PSO-XnB–Bagging_SS	10	0.932	0.933	0.01035	0.00327	17	9	<0.001
PSO-XnB–XGBoost_SS	10	0.936	0.935	0.00974	0.00308	15.55	9	<0.001
PSO-XnB–MS	10	0.971	0.972	0.00354	0.00112	N/A	N/A	N/A
PSO-XnB–RF_MS	10	0.729	0.732	0.01716	0.00543	44.49	9	<0.001
PSO-XnB–GB_MS	10	0.646	0.651	0.02473	0.00782	43.47	9	<0.001
PSO-XnB–Bagging_MS	10	0.828	0.831	0.03472	0.01098	12.37	9	<0.001
PSO-XnB–XGBoost_MS	10	0.861	0.866	0.04136	0.01308	8.17	9	<0.001
PSO-XnB–LS	10	0.983	0.984	0.00238	7.54E-04	N/A	N/A	N/A
PSO-XnB–RF _ LS	10	0.786	0.791	0.01742	0.00551	38.51	9	<0.001
PSO-XnB–GB _LS	10	0.275	0.281	0.03942	0.01247	57.28	9	<0.001
PSO-XnB–Bagging_LS	10	0.853	0.848	0.0695	0.02198	5.98	9	<0.001
PSO-XnB–XGBoost_LS	10	0.941	0.942	0.00416	0.00132	40.48	9	<0.001
PSO-XnB–ES	10	0.999	0.999	6.92E-04	2.19E-04	N/A	N/A	N/A
PSO-XnB–RF_ES	10	0.99	0.99	0.00592	0.00187	4.39	9	0.002
PSO-XnB–GB_ES	10	0.8	0.811	0.03639	0.01151	17.18	9	<0.001
PSO-XnB–Bagging_ES	10	0.885	0.886	0.0515	0.01629	7.01	9	<0.001
PSO-XnB–XGBoost_ES	10	0.992	0.992	0.00542	0.00171	4.49	9	0.002

**Figure 7:**
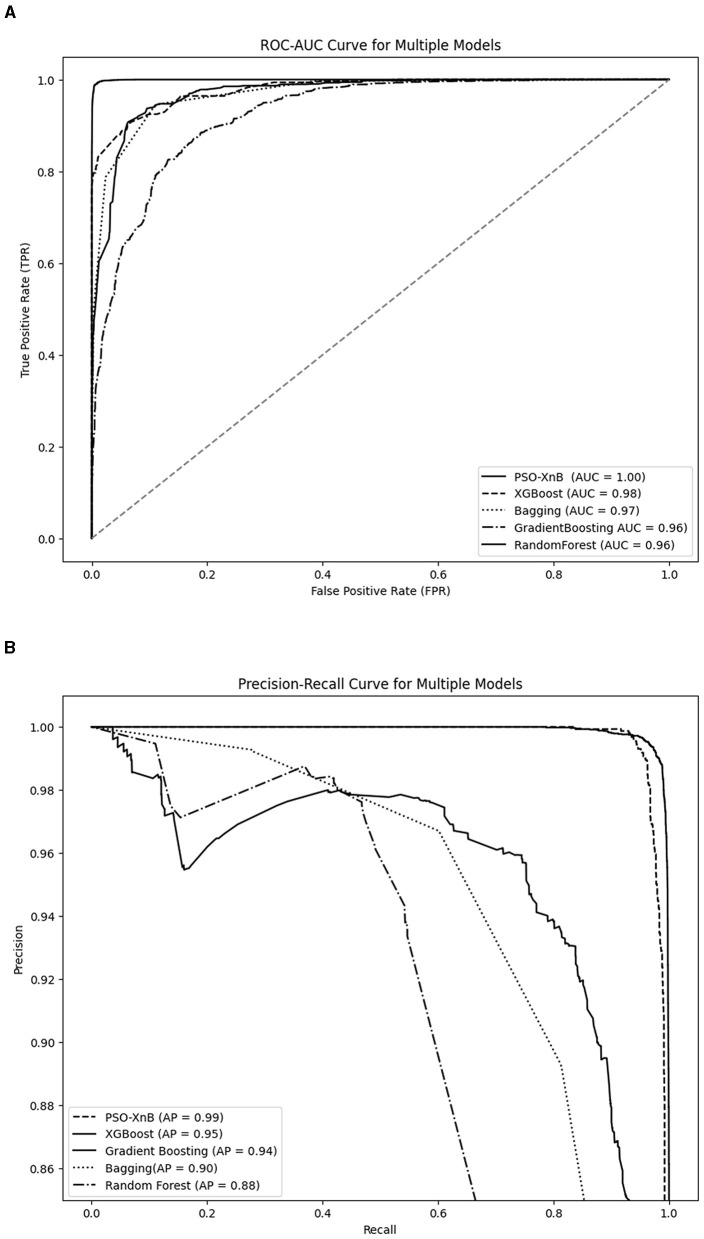
Performance evaluation graphs of PSO-XnB with other ensemble models. **(A)** ROC-AUC curve. **(B)** Precision recall curve.

Figure 7A compares ROC-AUC scores as percentages; the perfect score of 1.00 achieved by PSO-XnB represents a rate of 100% with no false positives across all thresholds. In contrast, the XGBoost and Bagging models scored AUC with 98% and 97%, covering 2% and 3% less ROC space than the PSO-XnB model. Similarly, both GB and RF models have an AUC of 96%, covering 4% of the ROC area compared to PSO-XnB. These percentages represent the varying capacities of each model to differentiate between categories. While examining the Precision-Recall curves in Figure 7B, it becomes evident that the PSO-XnB model establishes a standard with an Average Precision (AP) score of 99.65%. Relative to this, the XGBoost, GB, Bagging, and RF model shows a 4%, 5%, 10%, and 11% decrease in an area with an AP with 95.36%, 94.54%, 90.23%, and 88.92%, respectively. In practical terms, these percentages of AP reflect the degree to which each model's performance on precision and recall falls short of the near-perfect score achieved by the PSO-XnB. A lower AP score means the model is either less precise, less sensitive, or both, especially in predicting positive instances within imbalanced datasets (Jiang et al., 2020).

**Figure 8:**
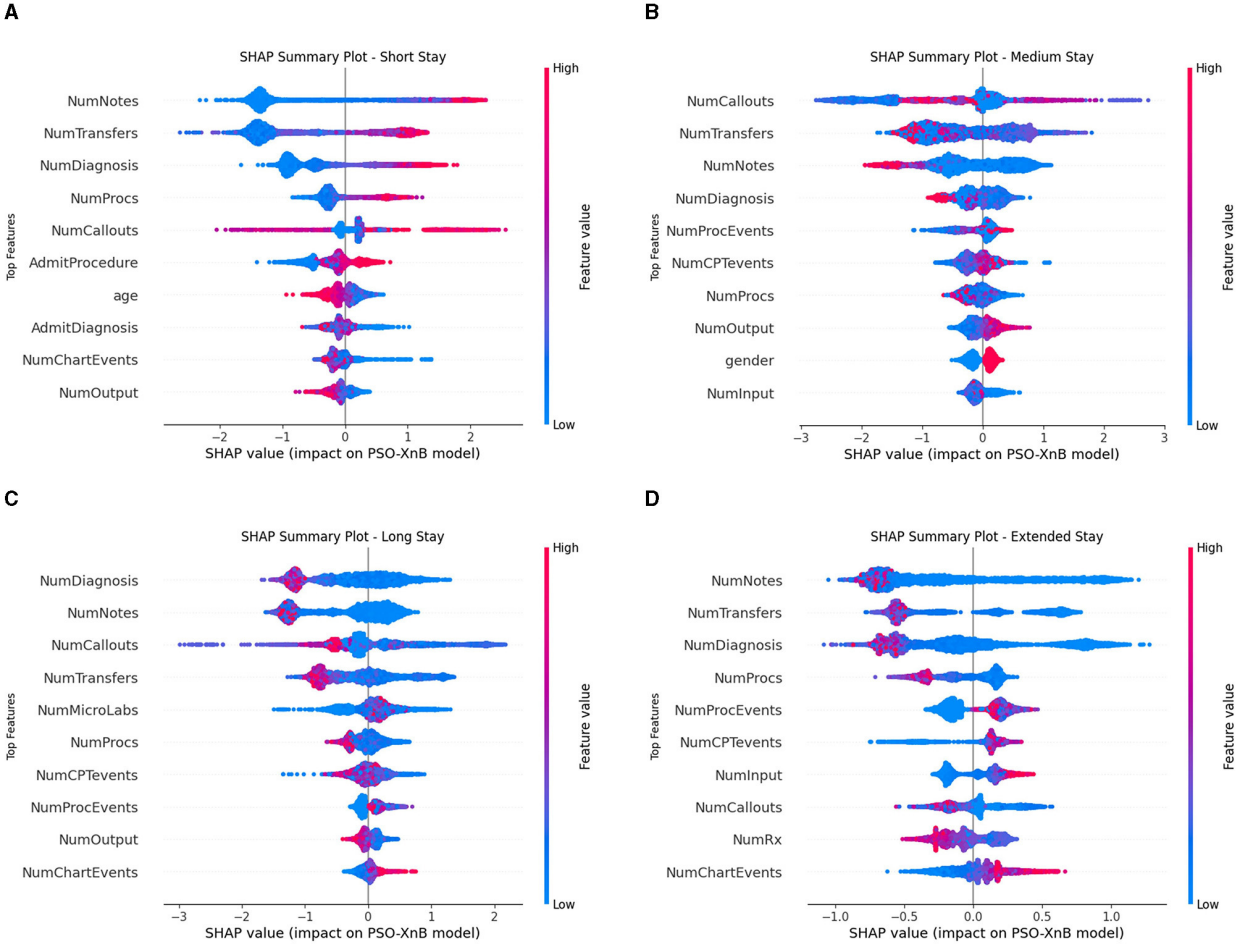
Feature impact model analysis using SHAP **(A)** Short stay. **(B)** Medium stay. **(C)** Long stay. **(D)** Extended stay.

The impact of the PSO-XnB model on features over four LOS classes is determined by SHAP analysis, as shown in Figure 8; the main two features impacting each class are discussed. In Figure 8A, the short stay shows the significance of NumNotes and NumTransfers features, indicating the number of medical notes and transfers between departments on patient records. These are important to predict short stays. Similarly, medium stay in Figure 8B shows NumCallouts determining the number of times staff were called to attend a patient as an impactful feature, along with NumTransfers. The features such as NumDiagnosis and NumNotes highly influence the long stay class in Figure 8C. These features show that the number of documented diagnoses and notes is important for long-stay predictions. Lastly, in Figure 8D, NumDiagnosis and NumProcs are influential for extended stays, where NumProcs determines the medical procedure that the patient has undergone. These high-impactful features on predictions are represented in red, whereas low features are depicted in blue. The insights into features impacting LOS classes can help clinicians optimize resource allocation. Also, hospitals can reduce complication risks and improve capabilities, enhancing patient outcomes.

From the literature review, some of the limitations observed were resolved with our proposed model PSO-XnB, such as overfitting, sensitivity of feature space dimensionality, and class imbalance. Although the PSO-XnB performs well in interpreting clinical data, its specific design is a limitation when considering its utility for various data analysis applications. Another limitation is that the optimization heavily depends on complex computational processes (Cao et al., 2023). This high computational demand could restrict the model's practical use, especially in healthcare environments. Addressing these challenges will broaden the model's applicability in decision-making and real-time processing, ensuring its utility in diverse healthcare scenarios. The comparison of our proposed model with other works from past literature is shown in Table 7.

**Table 7:** Comparison of the proposed model with previous literature.

**References**	**Models**	**No. of LOS classes**	**Accuracy**
**Our proposed model**	**PSO-XnB**	**4**	**98.8%**
Tavakolian et al. (2023)	GAOCNN	13	94.1%
Zou et al. (2023)	WGAN-GP	3	96.6%
González-Nóvoa et al. (2023)	XGBoost with Bayesian	1	92.0%
Hempel et al. (2023)	XGBoost With self-optimization	2	81.0%
Harerimana et al. (2021)	Hierarchical Attention Network	3	86.0%
Wang et al. (2020)	Random forest	1	92.3%
Gentimis et al. (2017)	Neural Network	2	79.8%

## 5 Conclusion

In this research, the Particle Swarm Optimized-Enhanced NeuroBoost (PSO-XnB) model showed significant achievement in predicting Hospital Length of Stay for CAD patients. This model combines particle swarm optimization with an eXtreme gradient-boosting model that utilizes a deep autoencoder technique for dimensionality reduction. Our proposed model PSO-XnB model showed outstanding performance efficiency with 98.8% accuracy. The model also scored the highest F1 score of all four LOS categories, compared with other ensemble models, with 0.99 for short stays, 0.98 for medium stays, 0.99 for long stays, and 1.00 for extended stays. This proposed model also demonstrated the high scores for other metrics such as precision, recall, and AUC. With accurate LOS predictions, healthcare services and patients can use our PSO-XnB model to take immediate initiatives. In the future, distributed and federated learning will be focused on observing the effects on classifier performance to reduce computational demands.

## Data availability statement

The original contributions presented in the study are included in the article/Supplementary material, further inquiries can be directed to the corresponding author.

## Author contributions

GM: Writing – review & editing, Writing – original draft, Visualization, Validation, Software, Resources, Project administration, Methodology, Investigation, Formal analysis, Data curation, Conceptualization. AS: Writing – review & editing, Supervision.

## Supplementary material

Supplementary Table S1 Performance metrics of PSO-XnB and Baseline traditional Ensemble models on ten-test splits.
